# Insights into HIV-1 Transmission Dynamics Using Routinely Collected Data in the Mid-Atlantic United States

**DOI:** 10.3390/v15010068

**Published:** 2022-12-25

**Authors:** Seble G. Kassaye, Zehava Grossman, Priyanka Vengurlekar, William Chai, Megan Wallace, Soo-Yon Rhee, William A. Meyer, Harvey W. Kaufman, Amanda Castel, Jeanne Jordan, Keith A. Crandall, Alisa Kang, Princy Kumar, David A. Katzenstein, Robert W. Shafer, Frank Maldarelli

**Affiliations:** 1 Department of Medicine, Georgetown University, Washington, DC 20057, USA; 2 HIV Dynamics and Replication Program, National Cancer Institute, Frederick, MD 21702, USA; 3 School of Public Health, Tel Aviv University, Tel Aviv 69978, Israel; 4 Warren Alpert Medical School, Brown University, Providence, RI 02912, USA; 5 Department of Medicine, Stanford University, Stanford, CA 94305, USA; 6 Quest Diagnostics, Secaucus, NJ 07094, USA; 7 Department of Epidemiology, George Washington University, Washington, DC 20052, USA; 8 Computational Biology Institute, George Washington University, Ashburn, VA 20147, USA

**Keywords:** molecular epidemiology, phylogenetic analysis, transmission networks, regional transmission dynamics, HIV drug resistance, clinical and phylogenetic data combined, contact tracing tool

## Abstract

Background: Molecular epidemiological approaches provide opportunities to characterize HIV transmission dynamics. We analyzed HIV sequences and virus load (VL) results obtained during routine clinical care, and individual’s zip-code location to determine utility of this approach. Methods: HIV-1 *pol* sequences aligned using ClustalW were subtyped using REGA. A maximum likelihood (ML) tree was generated using IQTree. Transmission clusters with ≤3% genetic distance (GD) and ≥90% bootstrap support were identified using ClusterPicker. We conducted Bayesian analysis using BEAST to confirm transmission clusters. The proportion of nucleotides with ambiguity ≤0.5% was considered indicative of early infection. Descriptive statistics were applied to characterize clusters and group comparisons were performed using chi-square or t-test. Results: Among 2775 adults with data from 2014–2015, 2589 (93%) had subtype B HIV-1, mean age was 44 years (SD 12.7), 66.4% were male, and 25% had nucleotide ambiguity ≤0.5. There were 456 individuals in 193 clusters: 149 dyads, 32 triads, and 12 groups with ≥ four individuals per cluster. More commonly in clusters were males than females, 349 (76.5%) vs. 107 (23.5%), *p* < 0.0001; younger individuals, 35.3 years (SD 12.1) vs. 44.7 (SD 12.3), *p* < 0.0001; and those with early HIV-1 infection by nucleotide ambiguity, 202/456 (44.3%) vs. 442/2133 (20.7%), *p* < 0.0001. Members of 43/193 (22.3%) of clusters included individuals in different jurisdictions. Clusters ≥ four individuals were similarly found using BEAST. HIV-1 viral load (VL) ≥3.0 log_10_ c/mL was most common among individuals in clusters ≥ four, 18/21, (85.7%) compared to 137/208 (65.8%) in clusters sized 2–3, and 927/1169 (79.3%) who were not in a cluster (*p* < 0.0001). Discussion: HIV sequence data obtained for HIV clinical management provide insights into regional transmission dynamics. Our findings demonstrate the additional utility of HIV-1 VL data in combination with phylogenetic inferences as an enhanced contact tracing tool to direct HIV treatment and prevention services. Trans-jurisdictional approaches are needed to optimize efforts to end the HIV epidemic.

## 1. Introduction

In February 2019, there was a call to end the HIV epidemic in the U.S. within a decade, using currently available treatment and prevention modalities [1]. Based on the current epidemiology in 2016–2017 of new HIV cases, 57 jurisdictions (48 counties, 7 states, Washington, District of Columbia. (DC), and Puerto Rico) were selected as over 50% of HIV diagnoses were made in these locations [2]. An estimated 14% of people living with HIV (PWH) were not aware of their condition and were thought to contribute approximately 38% of new transmissions with the remaining new infections arising from individuals with known HIV infection who were either not in care or not virally suppressed despite being in care [3]. Continued progress towards elimination of the HIV epidemic requires diagnosis of a higher percentage of PWH. Consistent early detection and treatment with durable viral suppression would reduce transmission [4]. This goal may be enhanced by implementing strategies to detect transmission clusters. Those can be reconstructed using HIV-1 sequence data. Expanded molecular epidemiologic investigation that also incorporates HIV-1 viral load (VL) may (i) identify undiagnosed PWH or those with suboptimal treatment with continued viremia who need antiretroviral treatment for their own health, but also to limit transmission to others [5,6,7]; and (ii) identify contacts at risk of infection for targeted provision of prophylactic antiretrovirals including appropriate pre- or post-exposure prophylaxis to prevent HIV acquisition [8,9,10,11].

Molecular epidemiologic approaches to characterize HIV transmission have been used in research and public health settings to describe local transmission dynamics [12,13,14]. Such studies have augmented our understanding of transmission dynamics previously based primarily on epidemiologic data alone. In 2014–2015, molecular epidemiologic approaches were critical to detect and monitor an HIV outbreak leading to 181 new HIV infections in southeastern Indiana related to drug injection [15]. The U.S. Centers for Disease Control and Prevention have since added a molecular surveillance component to routine public health activities to identify HIV outbreaks. Since then, this approach has been used to investigate outbreaks across the U.S. [16]. The approach focuses on identifying rapidly growing transmission clusters, with selection of a very low genetic distance threshold of 0.5% defining closely related viral sequences among individuals with recently diagnosed HIV. Only a small fraction of the new diagnoses invokes a public health investigation, with most investigations conducted by local health jurisdictions and limited to intra-state analyses.

DC has the highest HIV prevalence in the U.S. with an estimated 1.8% of the population living with HIV in 2019 [17]. DC residents have the highest estimated lifetime risk of acquiring HIV infection (1:39) followed in sixth place by neighboring Maryland (1:85) [18]. The adjacent geographic location of DC to Maryland and Virginia and the sharing of work force result in social and cultural interactions across borders, impacting the HIV epidemic. Using public health HIV surveillance data from these three contiguous jurisdictions, we found that approximately 21% of the PWH in the DC metropolitan area received care in more than one jurisdiction demonstrating the converging and overlapping epidemics in this high HIV transmission region [19]. Active movement of PWH in the metropolitan DC area is demonstrated with routine surveillance data that demonstrate outmigration of approximately 42% of PWH originally diagnosed in DC in 2019, and in-migration contributing 17% of the 12,408 PWH actively receiving care in DC [17]. Investigating HIV transmission dynamics in this region, we previously analyzed HIV *pol* sequence data from individuals who had enrolled in clinical or observational studies from early in the HIV pandemic [20,21,22]. Transmission clusters were common among men who have sex with men (MSM) and younger individuals. Individuals with evidence of recent infection based on low proportion of nucleotide ambiguity were more likely to belong in a transmission cluster [23,24]. 

In this study, we used HIV-1 sequences generated for clinical purposes along with limited demographic data, to characterize HIV transmission patterns in this high prevalence region. Our supposition is this approach would identify transmission clusters that traverse state borders, across this porous interconnected region, highlighting the importance of trans-jurisdictional surveillance to maximize its benefit in reducing HIV infection. We further postulate that laboratory surrogate markers for HIV care continuum metrics such as HIV-1 VL will provide an important discriminating factor to guide clinical and public health interventions.

## 2. Materials and Methods

### 2.1. Study Population

This is a retrospective molecular epidemiology study, using HIV sequencing data generated as part of routine clinical care from PWH receiving medical treatment in the mid-Atlantic region including DC, Maryland (MD) and Virginia (VA). HIV pol data sequenced using the Sanger method from 2008–2015 (protease, reverse transcriptase, and, when available, integrase) were obtained under a data use agreement with one national clinical laboratory. Additional information including age, sex, state, zip code, and year of service was available to the research team. A subset of individuals had quantitative HIV-1 RNA VL testing performed for routine clinical care at the Clinical Laboratory Improvement Amendments of 1988 (CLIA) certified national reference laboratory using a U.S. Food and Drug Administration cleared test method from Roche Molecular Systems (Branchburg, NJ, USA) with a lower limit of detection of 1.3 log_10_ copies/mL (c/mL). Data from samples obtained within a year of the sequencing date were included in this analysis. When zip code or state of residence were not provided, we used where medical service was provided as a location marker.

### 2.2. Sequence Alignment and Characterization

The earliest sequence available for each individual age ≥18 years, obtained during 2014–2015 were used. We aligned protease (Pr) and reverse transcriptase (RT) regions of HIV-1 pol sequences in Clustal-Omega version clustal-omega-1.2.1using HXB2 Pr-RT as the reference sequence spanning nucleotides 2252–3443 of the HXB2 reference sequence [25]. The Sierra pipeline to the Stanford HIV Database was used in sequence evaluation (subtype, gene coverage, and quality) and assessment of HIV-1 drug resistance [26]. The proportion of nucleotides with ambiguity ≤0.5% was used to indicate early or recent HIV infection within one year of infection [23,24].

### 2.3. Cluster Analyses

#### 2.3.1. Maximum Likelihood

A maximum-likelihood (ML) phylogenetic tree was constructed using IQ-TREE multicore [27] v1.5.0-beta with the general time-reversible (GTR) substitution model with invariable sites (I) and gamma (G) distribution (GTR+I+G) with 1000 bootstraps [28]. The FASTA sequences and the IQtree tree files were uploaded into ClusterPicker (v1.2.3) to identify sequences that were in clusters with ≥95% bootstrap node support for pre-specified genetic distance (GD) thresholds of 0.5%, 1.5%, 2.0%, 3.0% and 4.5% [27].

#### 2.3.2. Bayesian Analysis

To probe the likelihood that clusters actually represented local transmission networks, the specificity of cluster designation was assessed using a probability-based approach, the Bayesian evolutionary analysis sampling trees (BEAST) [29]. We identified sequences that were in groups of four or more at a GD ≤3% using the ML method. In order to verify the veracity and robustness of the transmission clusters we performed a blast search in the GenBank HIV sequence database for each sequence in this subset and selected the ten sequences most similar to each sequence in our data set [30]. Excluding duplicate sequences, sequences without dates, and those of significantly shorter length, we were left with 120 GenBank sequences. A total of 183 sequences comprising the study and GenBank sequences, with an additional subtype D reference sequence, were used for the BEAST analysis. The sequences were aligned as above. BEAST analysis was performed using the GTR+I+G substitution model, uncorrelated log-normal relaxed molecular clock [31], the parametric tree prior coalescent assumption of the Gaussian Markov random field (GMRF) skyride [32,33], and Markov chain Monte Carlo (MCMC) of iterations for 135 million states to achieve a posterior effective sample size of 302. Clusters of sequences that grouped together with a posterior probability nodal support of 95% or greater were identified and compared to the original clusters identified using the ML method to confirm the robustness of grouping.

#### 2.3.3. Network Analysis

Network analysis was performed to visually display the effect of different GD thresholds on the network composition. The aligned HIV sequence data were uploaded into HIV-TRACE [34] to implement the Tamura Nei 93 (TN93) substitution model that determines the pairwise distances between sequences [35]. The ambiguities between nucleotide pairs were resolved at an ambiguity fraction of 0.05 and the overlap was set at 500. The program generated a list of the nodes (represents sequences) and edges (connections between the nodes) used to derive network figures. The node and link attribute files were integrated with the remaining metadata and uploaded in the web-based HIV-TRACE visualization tool. Network figures were generated for GD thresholds of 0.5%, 1.5%, 2.0%, and 3.0%.

### 2.4. Statistical Analysis

We used R script to generate descriptive statistics to characterize the frequency and distribution of demographic and cluster data within the study population [36]. We conducted comparisons between groups using t-test and chi square tests for continuous and categorical data, respectively.

### 2.5. Ethical Considerations and Disclosures

This study was reviewed and approved by the Georgetown University Institutional Review Board under the terms of the data use agreement allowing the use of limited personal identifiers including zip code, with appropriate data security safeguards instituted by the Georgetown University Information Systems. Data from zip codes with fewer than 20 individuals were omitted to avoid unintended loss of privacy for these individuals. The terms of use limit access to these data to the study team and their delegates that include members of the national reference laboratory that generated these data. Publications resulting from this data set required review and approval by the national reference laboratory. 

## 3. Results

The analysis included 2775 individuals aged ≥18 years with genotype sequences available from 2014–2015 (Table 1). The mean age was 42.9 years (SD: 12.7, range: 18–79). The majority were males, (N = 1843, 66.4%). Residence was in MD for 2124 participants (76.5%); VA for 375 (13.5%); and DC for 242 (8.7%). Subtype B HIV-1 was the predominant subtype, 2589 (93.3%), of which 643/2589 (24.8%) sequences had the proportion of nucleotide ambiguity ≤0.5 suggestive of recent infection. 

### 3.1. Cluster Data

**Figure 1:**
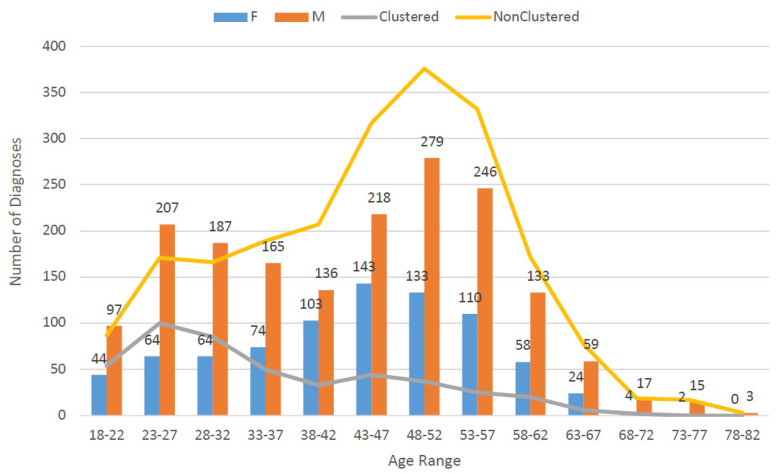
Distribution of individuals over clustered, non-clustered and sex by age group, genetic distance 3%. The bar graph demonstrates the frequency and age distribution of individuals in transmission clusters. The proportion of those in clusters (shown in gray) compared with those not in clusters (shown in yellow) is higher among younger individuals.

Among 2589 individuals with subtype B HIV-1, 456 (17.6%) aligned within 193 clusters using the ML method (GD 3%, 90% bootstrap support) (Table 2). The majority of clusters were dyads (149) and triads (32). There were 12 clusters of ≥4 individuals: 4 clusters of 4; 5 clusters of 5; and 1 cluster each with 6, 7, and 8 individuals. Proportionally, males were more likely to be in clusters than females, 349/1762 (19.8%) vs. 107/823 (13.0%), *p* < 0.0001. The age distribution of individuals based on cluster designation is demonstrated in Figure 1. Individuals in clusters were younger, with a mean age of 35.3 years (SD 12.1), compared to 44.7 years (SD 12.3) among those not in clusters (*p* < 0.0001). Individuals in clusters were more likely to have low proportion of nucleotides with ambiguity, ≤0.5%, suggestive of recent infection, with 201/456 (44.1%) having the low ambiguity score among those in clusters compared with 442/2133 (20.7%) among those not in clusters, *p* < 0.0001. The majority of individuals in clusters with low sequence ambiguity were males, 81% (163/201), and only 19% (38/201) were female. There was no statistically significant difference in the proportion of individuals in a cluster by state of residence. Of 193 clusters, 43 (22.3%) included individuals residing in different states: DC-MD in 20 clusters; MD-VA in 18 clusters; VA-DC in 4 clusters; and DC-MD-VA in 1 cluster.

When using a more stringent genetic distance threshold of 2%, the number of clusters decreased to a total of 109:88 dyads, 14 triads, four clusters of four, and three clusters of five individuals. Even fewer clusters (72) were identified using a genetic distance cutoff of 1.5%, with 59 dyads, 10 triads, and 3 clusters of 4 individuals. At the genetic distance threshold of 0.5%, only 13 dyads and 1 triad were identified. 

### 3.2. BEAST Analysis

**Figure 2:**
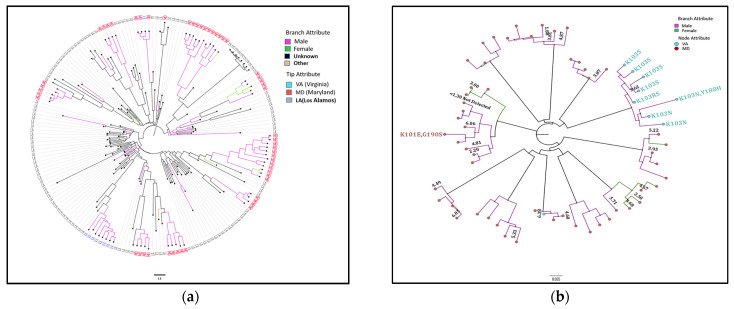
Phylogenetic trees of sequences in clusters with ≥4 members. (**a**). Study population sequences and closest related sequences from GenBank. Bayesian phylogenetic tree of 62 subtype B HIV-1 sequences that were identified in clusters size four or more based on GD 3% using the maximal likelihood method were included alongside the most closely related sequences from the GenBank HIV Sequence database at Los Alamos (labeled “LA”). Bayesian evolutionary analysis sampling trees [29] analysis was performed using the GTR+I+G substitution model, uncorrelated log-normal relaxed molecular clock, tree coalescent assumption of the Gaussian Markov random field (GMRF) skyride [31,32,33], and run of 135 million states to achieve an effective sample size of 302. The cluster relationships were maintained with high posterior probability of ≥95% when analyzed using this BEAST approach. Male participants are indicated in purple and females indicated in green, and sequences from Los Alamos indicated in black. (**b**). Cluster characteristics: HIV-1 drug resistance mutations and viral load. HIV-1 viral load data are written in black with the corresponding sequence when available. Among the 21 individuals with HIV-1 VL data available, 18 (85.7%) had levels ≥3 log10 c/mL. Four HIV-1 drug resistance associated mutations were identified in two clusters including the resistance mutations Y188H, K101E, K103N/S, and G190S. One cluster had multiple cluster members with drug resistance mutations.

All the clustered sequences with ≥4 members found using the maximal likelihood approach with a genetic distance threshold of 3% remained in clusters, with posterior probability support ≥0.95 (Figure 2a,b) when analyzed using BEAST. Twenty-one out of the sixty-two individuals had HIV-1 VL available, and 18/21 (85.7%) had HIV-1 VL ≥ 3.0 log_10_ c/mL. There was evidence of transmitted K103N/K103S drug resistance mutations within a large transmission cluster as shown in Figure 2b, with the resistance mutation noted among multiple members of the cluster. The range and frequency of major HIV-1 resistance mutations are shown in Figure S1.

### 3.3. Network Analysis

**Figure 3:**
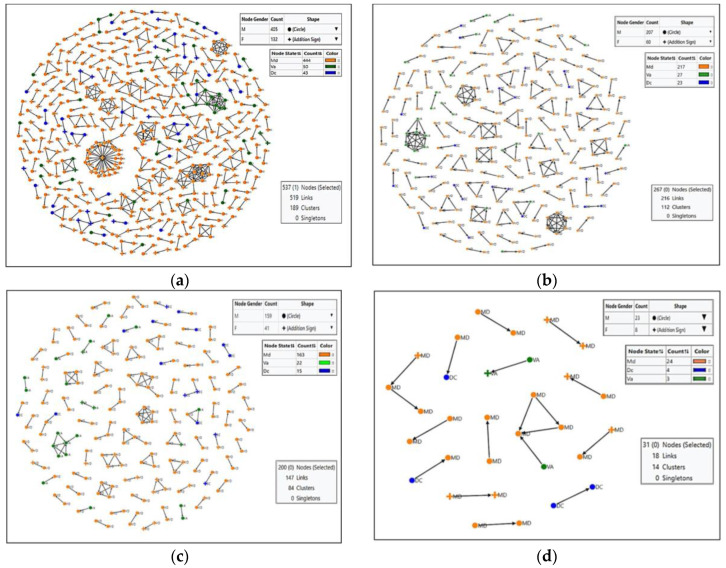
HIV-TRACE Transmission Networks by Genetic Distance. Cluster networks configured in HIV-TRACE from HIV pol sequences are inferred using genetic distances between sequences. Cluster networks demonstrate decreasing linkages with lower genetic distance cutoffs ((**a**) ≤3%; (**b**) ≤2%; (**c**) ≤1.5%; and (**d**) ≤0.5%), although multijurisdictional networks are identified at all genetic distance cutoffs. Using the less restrictive genetic distance cutoff of 3% provides greater detail and the resulting network visualization provides context and highlights connections that more completely represent potentially important HIV-1 transmission dynamics.

Using HIV-TRACE we identified 519 nodes linked to at least one other node at GD 3.0% (Figure 3a–d). A total of 189 clusters were formed, of which the largest had 33 nodes with 46 connected links at a mean genetic distance of 2.8% and 1.39 links per node. The second-largest dense cluster consisted of 15 nodes making 38 links with a higher transmission speed of 2.53 links per node. The male transmission clusters comprised 66% of identified networks and the female clusters were 10.5%. Recent infection based on the proportion of nucleotide ambiguity ≤0.5% identified a higher number of nodes from 2014 than from 2015. Networks spanning more than one jurisdiction were identified in 75 nodes comprising 14% of individuals identified to be in a transmission cluster.

### 3.4. Transmission Clusters and Viremia

HIV-1 VL was available for 54% (N = 1394) of the study population (Table 3). The majority, 78% (1091/1394) had VL ≥3.0 log_10_ copies/milliliter (c/mL); 3.5% (49/1394) had VL 2.7–3.0 log_10_ c/mL; 17.4% (242/1394) had VL 1.3–2.7 log_10_ c/mL, and 0.01% (12/1394) individuals had VL <1.3 log_10_ c/mL. The median VL was 4.4 log_10_ c/mL (IQR 25–75%: 3.2–4.99). The quantitative VL differed by cluster size with the highest VL among those in clusters of four or greater, median VL 4.6 log_10_ c/mL, compared with 3.9 c/mL log_10_ for those in clusters of 2 and 3, and 4.4 log_10_ c/mL among those not in a cluster. The proportion of individuals with HIV-1 VL ≥3.0 log_10_ c/mL varied among different-sized cluster groups and was highest in the group with cluster size ≥4, 85.7% (18/21); 65.8% (137/208) among those in cluster size 2–3; and 79.3% (927/1169) among those individuals not in a cluster (*p* < 0.0001).

## 4. Discussion

This collaborative project was designed to determine the utility of regional sequence and HIV-1 VL data generated as part of clinical care for the characterization of HIV transmission patterns, given the highly interconnected mid-Atlantic metropolitan DC area. An important element of our research is the public-private-academic partnership that allowed us to access this representative regional dataset generated in the course of routine clinical care with multi-jurisdictional representation under a legally binding data sharing agreement. Enrolling such a comprehensive cohort to obtain sequence and laboratory data for research purposes only would have been challenging and costly. With the safeguards that we have placed both in the design and implementation of the study to protect individual’s privacy, we demonstrate the feasibility and utility of such collaborations. Our findings of the relative frequency of trans-jurisdictional transmission clusters in this highly interconnected region highlights the need to overcome restrictions in data sharing based on traditional jurisdictional borders, regional or national. Such restrictions limit collaborations and innovative strategies that span jurisdictions are essential to adequately respond to the call to end the HIV epidemic.

We demonstrate the high fidelity of rapid maximal likelihood phylogenetic approaches for identifying transmission clusters, further validated with the more time intensive probability-based sequence and network analyses. Overall, we rarely identified large transmission clusters, with most clusters containing two or three sequences in this convenience sample of sequences. There were few transmission clusters with size 4 or greater. This is likely the result of the sampling framework that was not focused exclusively on newly diagnosed PWH, but rather included all individuals for whom HIV sequence data were generated for clinical management, including those with chronic HIV-1 infection. In addition to MSM and younger individuals, we also identified a subset of individuals in their fourth or higher decades of life, including women, in transmission clusters. Interventions to end the HIV epidemic in the region will need targeting also these important and often missed sub-populations that remain at risk for HIV-1 infection.

Evidence supporting the validity of rapid maximal likelihood based phylogenetic methods shows that large data sets can be analyzed to support contact tracing within relatively short periods of time, with computational capacity that is generally available in many public health settings. In this context, more recent uses of HIV sequence data have emerged that focus on identifying new HIV outbreaks. These projects have primarily used very narrow genetic distance thresholds of 0.5–1.5% [13,14]. While this may be appropriate for identifying emerging large outbreaks, these cutoffs yield o reduced case numbers for investigation when applied to our regional dataset, with only fourteen clusters (thirteen dyads and one triad) fitting the most stringent metric. Most individuals found in larger clusters, identified using the genetic distance threshold of 3% and verified using a network and probability based phylogenetic analysis with BEAST, would have been missed. A significant proportion of individuals with evidence of linked transmission in our analyses demonstrated very low proportion of nucleotide ambiguity, suggesting that some of these infections indeed could have been more recently acquired. The relatedness of individuals within clusters using a less stringent genetic distance cutoff could be epidemiologically important in a mature epidemic such as in the mid-Atlantic region, where linked transmissions may span decades. The consistent findings despite using different methods of inference to determine sequence relatedness demonstrate justifies using a 3% genetic distance threshold to robustly identify relevant transmission clusters. The persistence of the identified clusters after the addition of the most closely related sequences from GenBank suggests supports likely epidemiologic linkages among the persons from whom sequences were obtained. While use of currently accepted genetic distance thresholds in public health molecular surveillance programs are set to identify emerging outbreaks, we demonstrated how these thresholds would miss identifying epidemiologically important targets for intervention in the context of an established generalized epidemic. Relaxing the stringency beyond 3%, though, appeared counter-productive in our case as it may result in artifactual linkages, a finding that has previously been reported by others [13].

The addition of selected laboratory testing such as HIV-1 VL further increases the potential utility of molecular sequence data. HIV-1 VL is an important marker of linkage, engagement and retention in care in the context of the HIV care continuum [37]. The most recent report indicates that 57% of the estimated 1.2 million individuals with HIV in the U.S. had viral suppression [38]. Ideally, all individuals with detectable viral load would benefit from interventions to support linkage to care. We demonstrate how overlaying molecular epidemiology with HIV-1 VL data allows for further risk stratification such that individuals with evidence for recent infection based on sequence ambiguity, cluster membership, and high HIV-1 VL would be preferentially targeted for support services if resources are constrained. Natural history studies have demonstrated HIV-1 VL of approximately 3 log_10_ c/mL as the threshold for transmission risk, with escalating VL associated with increasing transmission risk [39]. More recent data have identified that ART use with durable viral suppression decreases risk of transmission [5,6,7]. The relatively high proportion of individuals within clusters with HIV-1 VL ≥ 3log_10_ copies/mL highlights the need for targeted and effective ART programs. While all individuals should receive ART for their own health, those who are in transmission clusters with high VL could be prioritized for adherence support and interventions to halt further transmission. Individuals who are in dyads and triads are often not considered high value targets for intervention and contact tracing but are more frequently identified than the larger transmission networks that are currently the target of public health interventions [40]. The addition of HIV-1 VL data to transmission cluster data allows one to discern which individuals are biologically more likely to transmit and hence should be prioritized for supportive intervention. Such an approach may allow us to break through the current plateau in new HIV-1 diagnoses towards the goal of ending the HIV epidemic.

Earlier molecular epidemiology studies in clinical settings have demonstrated the decreased transmission risk with antiretroviral treatment and viral suppression [41]. We demonstrate how molecular epidemiologic elements can bolster efforts to identify individuals with potential for transmission at the local population level, using routinely collected clinical management laboratory data. Ideally, all individuals with viremia should receive interventions to support engagement in care. In the absence of unlimited resources, however, priority could be directed to those with elevated viremia for whom there is molecular epidemiologic support for risk for transmission [42], thereby providing an additional incentive for intervening both for the sake of the individual’s health and to reduce transmission to others. Such active monitoring with expanded use of molecular data in conjunction with clinical metadata such as HIV-1 VL would contextualize and refine assessments of transmission risk [42]. Individuals in larger cluster sizes who remain viremic have greater potential to transmit HIV, risk that could be effectively mitigated with appropriate outreach to ensure engagement in care and effective suppressive antiretroviral therapy. Discovery of risk before transmission and successful mitigation represents the holy grail of infectious disease control, and successful implementation would serve to accelerate the progress towards ending the HIV epidemic.

One of the limitations of our study is the sampling frame only covers an estimated quarter of data that are generated from individuals in the region. While the lower sampling density may decrease the number of transmission clusters that are identified due to missing data, our findings suggest that the identified clusters are robust. The persistence of the identified clusters after the addition of the most closely related sequences from GenBank suggests that the inferred relationships were not simply due to sampling bias. Our data are limited to PWH receiving care and does not capture the important subset of PWH who are not in care. Although having near complete sampling from the affected community would result in finding more transmission clusters, even this subset yields important information with actionable findings, of the kind pivotal to ending the HIV epidemic efforts. While individual data related to social contacts was not available in our data set, partner information is often under-reported [43]. The dataset does not include ART histories and thus limits full characterization of the HIV care-continuum for the studied population.

Lastly, there has been a mixed response to using molecular epidemiology to characterize transmission dynamics both in the scientific community and among advocacy groups [44,45,46,47]. It remains critical to assess any presumed negative consequences. Our analysis validates the utility of genotypic and viral load data even in the absence of detailed epidemiologic data and may be an important adjunctive public health tool in areas where concerns related to stigma and marginalization may limit the engagement of people living with HIV [48].

## 5. Summary

Optimal use of molecular genotypic data should include more relaxed genetic distance cutoffs to identify putative transmission clusters. These data should be analyzed in the context of additional routine clinical and laboratory data that are collected during the implementation and monitoring of standard of care treatment and prevention programs. Such an approach would provide context and identify opportunities for intervention to guide care teams to provide differentiated care services and optimize resource allocation and utilization. Only through bold and integrated interventions will we reach the next milestone towards ending the HIV epidemic.

## Figures and Tables

**Table 1:** Cohort Socio-demographic and HIV-1 Characteristics, 2014–2015.

		Subtype B HIV-1 N = 2589	Non-B Subtypes N = 186	Total N = 2775
Gender	Female	823	105	928
		(31.8%)	(56.5%)	(33.4%)
	Male	1762	81	1843
		(68.1%)	(43.5%)	(66.4%)
	Not Reported	4	0	4
		(0.15%)		(0.14%)
State	DC	232	10	242
		(9%)	(5.4%)	(8.7%)
	MD	1988	136	2124
		(76.8%)	(73.1%)	(76.5%)
	VA	338	37	375
		(13.1%)	(19.9%)	(13.5%)
	Not Reported	31	3	34
		(1.2%)	(1.6%)	(1.2%)
Age	18–37 years	902	64	966
		(34.8%)	(34.4%)	(34.8%)
	38–52 years	1014	91	1105
		(39.1%)	(48.9%)	(39.8%)
	≥53 years	673	31	704
		(26.0%)	(16.6%)	(25.3%)
Ambiguity (Amb) *	Amb ≤ 0.5	643	29	672
		(24.8%)	(15.6%)	(24.2%)
	Amb 0.5–0.99	443	26	469
		(17.1%)	(13.9%)	(16.9%)
	Amb ≥ 1	1503	130	1633
		(58.1%)	(69.8%)	(58.8%)
Clustered Genetic Distance 3.0%	Female	107	1	108
		(4.1%)	(0.5%)	(3.9%)
	Male	349	3	352
		(13.5%)	(1.6%)	(12.7%)
	Total	456	4	460
		(17.6%)	(2.2%)	(16.6%)

* Ambiguity (Amb)—proportion of nucleotides within the sequence with ambiguity. Percentages may not add up to 100% due to rounding.

**Table 2:** Comparative characteristics of individuals with subtype B HIV-1 identified within and outside clusters with a genetic distance threshold of 3%.

		Clustered N = 456	Non-Clustered N = 2133	Total N = 2589	Chi-Square
Gender	Female	107	716	823	*p* < 0.0001
		(23.5%)	(33.6%)	(31.8%)	
	Male	349	1413	1762	
		(76.5%)	(66.2%)	(68.1%)	
	Mean (SD)	35.3 (12.1)	44.7 (12.3)	43 (12.8)	*p* < 0.0001
Age	18–37 years	289	613	902	*p* < 0.00001
		(63.4%)	(28.7%)	(34.8%)	
	38–52 years	114	900	1014	
		(25.0%)	(42.2%)	(39.2%)	
	≥53 years	53	620	673	
		(11.6%)	(29.1%)	(26.0%)	
Ambiguity (Amb) Score	Amb ≤ 0.5	202	442	644	*p* < 0.00001
		(44.3%)	(20.7%)	(24.9%)	
	Amb 0.5–0.99	99	343	442	
		(21.7%)	(16.1%)	(17.1%)	
	Amb ≥ 1	155	1348	1503	
		(34.0%)	(63.2%)	(58.1%)	
State	DC	36	196	232	*p* = 0.09 (NS)
		(7.9%)	(9.2%)	(9%)	
	MD	370	1618	1988	
		(81.1%)	(75.9%)	(76.8%)	
	VA	48	290	338	
		(10.5%)	(13.6%)	(13.1%)	
	NR	2	29	31	
		(0.4%)	(1.4%)	(1.2%)	

Percentages may not add up to 100% due to rounding.

**Table 3:** Relationship between HIV-1 viral load (VL) and transmission cluster size (genetic distance 3%).

Cluster Data		HIV Viral Load (VL)		
	Number of HIV VL Tests N = 1394	Median in log_10_ Copies/mL (IQR 25–75%)	≥3.0 log_10_ c/mL	<3.0 log_10_ c/mL
Overall HIV VL	1394	4.4 (3.2–4.99)	1091 (78.3%)	303 (21.7%)
Cluster size ≥ 4 (N = 62)	21	4.6 (3.8–5.2)	18 (85.7%)	3 (14.3%)
Cluster size 3 (N = 96)	47	3.1 (2.3–4.6)	26 (55.3%)	21 (44.7%)
Cluster size 2 (N = 298)	161	4.0 (2.1–4.8)	111 (68.9%)	50 (31.1%)
Cluster size 2 and 3 (N = 394)	208	3.9 (2.2–4.8)	137 (65.9%)	71 (34.1%)
Not in clusters (N = 2133)	1169	4.4 (3.3–5.0)	927 (79.3%)	242 (20.7%)

## Data Availability

The data were made available to the study team through a Data Licensing and Services agreement between QUEST Diagnostics and Georgetown University and does not permit transfer of the data. The national reference laboratory has rights to the data.

## Supplementary Materials

The following supporting information can be downloaded at: https://www.mdpi.com/article/10.3390/v15010068/s1, Figure S1: Frequency of major reverse transcriptase and protease mutations in B and Non-B Subtype HIV-1.
