# Supplementary material for: Insights into HIV-1 Transmission Dynamics Using Routinely Collected Data in the Mid-Atlantic United States
Source: Viruses. 2022 Dec 25;15(1):68. doi: 10.3390/v15010068 (PMC9863702; doi:10.3390/v15010068)
Supplement: Supplementary file 1 [file viruses-15-00068-s001.zip › viruses-2003604-supplementary.pdf]

viruses

MDPI

Figure S1. Frequency of major reverse transcriptase and protease mutations in B and Non-B Subtype HIV-1.

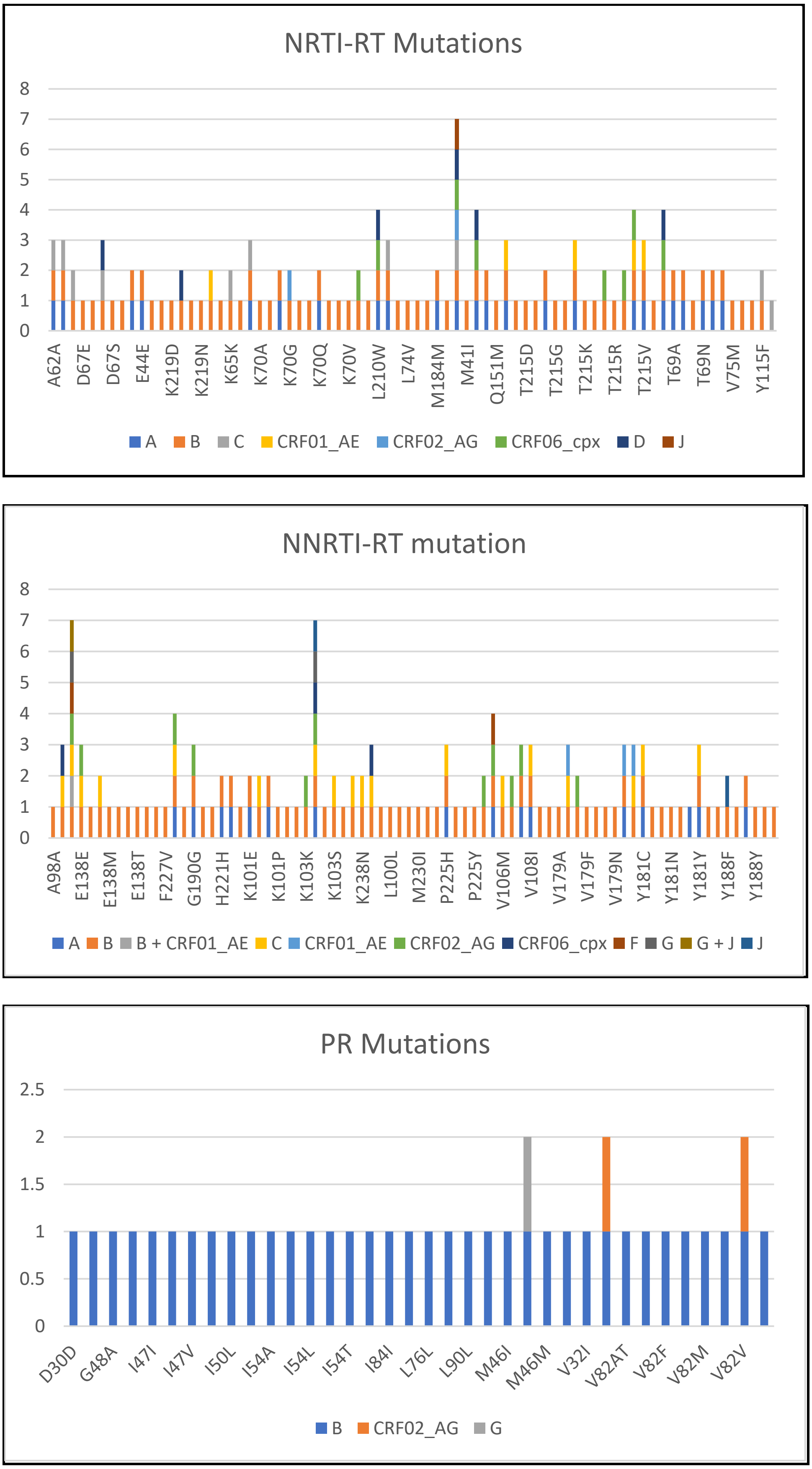

NRTI: nucleoside reverse transcriptase inhibitor.

NNRTI: non-nucleoside reverse transcriptase inhibitor.

PR: protease inhibitor.

RT: Reverse transcriptase.
